# To what extent have national learning objectives in undergraduate medical education been achieved? A cross-sectional study of primary care residents

**DOI:** 10.3205/zma001762

**Published:** 2025-06-16

**Authors:** Dorothea Dehnen, Kristina Flägel, Dorothea Wild, Jost Steinhäuser

**Affiliations:** 1 University of Duisburg-Essen, Medical Faculty, Institute of General Practice, Essen, Germany; 2 University Hospital Schleswig-Holstein, Institute of Family Medicine, Lübeck, Germany; 3 University of Bonn, University Hospital Bonn, Institute of General Practice and Family Medicine, Bonn, Germany

**Keywords:** practical clinical skills, competency level, NKLM, medical education, competency-based education, postgraduate training

## Abstract

**Aim:**

2021 saw the publication of the new version (2.0) of the “National Competency-based Catalogue of Learning Objectives (NKLM) in Undergraduate Medicine”, which in future will be closely linked to the German medical licensing regulations (ÄApprO). Included in the updated catalogue are specifically defined competencies concerning practical clinical skills. We aimed to determine how residents perceive their competency level to perform selected practical clinical skills in the NKLM 2.0.

**Method:**

In June 2022, all 593 medical residents registered at the competence centers for postgraduate training in primary care in North Rhine, Westphalia-Lippe and Schleswig-Holstein were invited to participate in an online survey. The participants were asked to retrospectively self-assess (1) their proficiency level (5-point Likert scale) at the beginning of their postgraduate training in regard to 36 practical clinical skills from the NKLM 2.0 and (2) where they had gained proficiency in those skills. Of the 164 participating residents, the main focus was on those who had been licensed for less than five years.

**Results:**

The responses of 47 residents were analyzed. For 29 skills, at least 20% of the participants stated that these had not been proficiently mastered at the start of postgraduate training; e.g., examining the spine of an adult (19.6%) or the skin (37.0%). For 14 skills – such as examining female and male genitalia and those of newborns and infants – more than 50% of the participants indicated that they had not been able to perform these skills.

**Conclusion:**

The results provide initial evidence that discrepancies may exist between the level of desired competency by the end of undergraduate medical education, as specified by the NKLM, and the level of proficiency actually achieved in terms of practical clinical skills. More teaching and feedback methods may need to be established to impart these skills during undergraduate medical education and to integrate these skills into complex clinical contexts.

## Zusammenfassung

**Zielsetzung:**

2021 wurde die aktualisierte Version (2.0) des zukünftig eng mit der Approbationsordnung verbundenen Nationalen kompetenzbasierten Lernzielkatalogs Medizin (NKLM) herausgegeben. In diesem werden auch zu klinisch-praktischen Fertigkeiten konkret zu erreichende Kompetenzen angegeben. Ziel dieser Studie war es zu erheben, mit welchem Kompetenzniveau Ärzt:innen in Weiterbildung (ÄiW) ausgewählte im NKLM 2.0 aufgeführte klinisch-praktische Fertigkeiten in der Selbsteinschätzung ausführen können.

**Methodik:**

Im Juni 2022 wurden 593 in den Kompetenzzentren Weiterbildung Allgemeinmedizin Nordrhein, Westfalen-Lippe und Schleswig-Holstein registrierte ÄiW gebeten, an einer Online-Umfrage teilzunehmen. Die Teilnehmenden (TN) sollten, in der Rückschau auf den Beginn der Weiterbildung, ihr damaliges Kompetenzniveau (5-stufige Likert-Skala) von 36 klinisch-praktischen Fertigkeiten aus dem NKLM 2.0 und den Ort des Kompetenzerwerbs angeben. Von den 164 teilnehmenden ÄiW wurde der Fokus auf die TN gelegt, deren Approbation maximal fünf Jahre zurücklag.

**Ergebnisse:**

Ausgewertet wurden die Antworten von 47 ÄiW. Bei 29 Fertigkeiten gaben mindestens 20% der TN an, diese zu Beginn der Weiterbildung nicht beherrscht zu haben, z. B. die Untersuchung der Wirbelsäule eines Erwachsenen (19,6%) oder der Haut (37,0%). Bei 14 Fertigkeiten wie beispielsweise der Untersuchung des weiblichen und männlichen Genitals sowie von Neugeborenen und Säuglingen haben >50% der TN mitgeteilt, diese nicht beherrscht zu haben.

**Schlussfolgerung:**

Die Ergebnisse liefern erste Hinweise, dass Diskrepanzen zwischen dem zukünftig nach dem NKLM zum Ende des Studiums zu erreichenden und dem aktuell erlebten Kompetenzniveau klinisch-praktischer Fertigkeiten bestehen könnten. Damit zeigt sich ein Bedarf einer verstärkten Etablierung von Lehr- und Feedbackmethoden zur Vermittlung dieser Fertigkeiten im Medizinstudium und deren Einbettung in komplexe-klinische Kontexte.

## 1. Introduction

The “Master Plan for Medical Study 2020”, adopted in 2017 by the German federal and state ministries of health and sciences, aims to enable more practice-based learning in medical education and give general practice more importance [1]. Further details are provided in the Federal Ministry of Health's (BMG) current draft legislation on medical education reform [2]. The Master Plan envisions, among other things, a longitudinal curriculum with (primary care) rotations that sequentially build upon each other. The curriculum should place a stronger focus on practical relevance of learned content, integrating theoretical and clinical competencies from the first years of study and conclude with a mandatory three months rotation in the final year, spent in an outpatient practice participating in the statutory health insurance [2], [3].

In order to promote independent and responsible medical practice, competency-based education and the “National Competency-based Catalogue of Learning Objectives (NKLM) in Undergraduate Medical Education” form the basis of a future “core curriculum” [2], [4]. The NKLM, based in part on the Canadian Medical Education Directives for Specialists (CanMEDS) Framework [5], [6], was first published in 2015 with the aim of improving the quality of medical education. It defines the competencies in which every physician should be proficient upon graduation from medical school. These competencies comprise not just knowledge and skills but also much broader learning objectives such as attitudes, scholarly abilities, and “soft skills” [4]. NKLM 2.0 – a further development of the first version of the NKLM – was presented in 2021 [https://nklm.de/zend/objective/list/orderBy/@objectivePosition/studiengang/Info] and is meant to define content areas for medical licensing examination in future. Since NKLM 2.0 objectives may become part of medical licensing regulations (ÄApprO) [2], the relevance its learning objectives formulated will increase significantly. This makes it even more important to determine whether physicians have attained the NKLM learning objectives at the specified standard of proficiency at the beginning of postgraduate training.

From previous surveys it is known that German graduates often have good theoretical knowledge, but lack practical application [7], [8]. In one study, the majority of the surveyed physician trainees stated that they were unable to independently perform general practice procedures such as otoscopy, bladder catheter insertion or cardiac stress test (ergometry) [8]. So far, no nation-wide survey of medical residents in Germany focusing on the NKLM 2.0 learning objectives has been performed; however, results would be particularly meaningful in regard to the further development of teaching methods to achieve the goals of the NKLM.

To optimize the quality and efficiency of postgraduate training in General Medicine, competence centers have been established across Germany since 2018 based on Section 75a of Book 5 of the German Social Code [9], [10]. At these centers, practice-based material is taught interactively, including in seminar programs for medical residents, e.g., through case-based, small-group assignments and training [11], [12], [13], [14]. Competence centers could counteract possible learning deficiencies.

Given the future relevance of the NKLM 2.0 learning objectives for medical education, the aim of this study was to investigate the extent to which physicians currently undergoing postgraduate training and who were not educated according to NKLM 2.0, self-assess their level of competency at the beginning of their postgraduate training to independently perform practical clinical skills [https://nklm.de/zend/objective/list/orderBy/@objectivePosition/studiengang/PF2/zeitsemester/2021/fachsemester/VIII.7.%20Klinisch-praktische%20Fertigkeiten] in the NKLM 2.0.

## 2. Methods

### 2.1. Questionnaire

Thirty-six practical clinical skills from the NKLM 2.0 relevant to primary care with a target competency level of 3a *(perform and demonstrate under guidance)* or 3b *(perform independently in a manner appropriate to the situation with knowledge of the consequences)* [https://nklm.de/zend/objective/list/orderBy/@objectivePosition/studiengang/PF2/zeitsemester/2021/fachsemester/VIII.7.%20Klinisch-praktische%20Fertigkeiten] [15] were selected for the survey in a consensus procedure involving one specialist in general practice and two medical residents. Questions about sociodemographic details were also added, along with 12 questions taken from a preliminary study [16] that were not included in the following analysis.

The primary care residents were asked to recall their level of competency when they first began to practice by assessing how proficient they were at each practical clinical skill. If the response indicated a skill had been mastered (regardless of competency level), respondents were then asked where this skill had been learned – during undergraduate medical study (including clinical electives and the final practical year) or elsewhere, e.g., previous professional training. The self-assessment of the competency level was captured on a 5-point Likert scale ranging from “I was unable to do this” to “I was able to teach someone else how to do this” [8], [17].

A pilot was conducted with five residents and a specialist in general practice to further adapt the questionnaire. The final version of the questionnaire can be found in the attachment 1 .

### 2.2. Recruitment

From June to July 2022, a link to an online survey was sent by email to all of the residents (n=593) pursuing specialist training in primary care who were registered at the postgraduate competency centers in North Rhine (n=236), Westphalia-Lippe (n=145) and Schleswig-Holstein (n=212). Reminders were sent by email after one and three weeks. To boost participation in the anonymous online survey at the North Rhine competency center, invitations via QR codes were also given out on three seminar days in the fall of 2022. The online survey was conducted using LimeSurvey (Limesurvey GmbH, Hamburg, Germany).

### 2.3. Data analysis

The analysis of the data was done using SPSS 27 (IBM SPSS Inc., Chicago, IL, USA). The data on the self-assessed competency levels for the practical clinical skills and the location where the competency was acquired were descriptively analyzed. We focused on participants who had been licensed within the past 5 years.

In a sub-analysis, cross-tabulation and Pearson's chi-squared test were used to check if the results for the residents whose licensure within five years significantly differed from the residents who had studied under the same licensure regulations (ÄApprO of 27 Feb. 2002, taking effect on 1 Oct. 2003) [https://www.gesetze-im-internet.de/_appro_2002/index.html] and whose medical licenses had been issued more than six but no earlier than 13 years ago (assuming six years of formal study in compliance with a license granted in 2009). The significance level was adjusted using the Bonferroni correction for multiple tests [18].

### 2.4. Ethics

Ethical approval was given by the Medical Faculty of the University of Duisburg-Essen *(22-10505-BO, 18.02.2022)*. Furthermore, an affirmative second vote was received from the Medical Faculties of the University of Bonn *(216/20, 27.4.2022 to the Amendment of 13.3.2022)* and the University of Lübeck *(22-124, 25.03.2022)*.

## 3. Results

### 3.1. Sample description

A total of 206 residents (34.7%) opened the survey, 164 residents (27.6%) participated. Overall, 47 residents (license ≤5 yr.) were included in the main analysis and 44 residents (license 6-13 yr.) in the sub-analysis. A total of 73 residents were excluded because they had studied abroad (n=12) and/or already had completed specialty training in another specialty (n=27) and/or their medical license was older than 13 years (n=20) and/or they had not stated the year of licensure (n=27).

The 47 participants were on average 34 years old (min. 26; max. 53), the majority were female (66.0%), in the 3^rd^ (23.4%), 4th (29.8%) or 5^th^ year of residency training (21.3%), and working at a medical practice (74.5%). A total of 25.5% of the residents were affiliated with the competence center in North Rhine, 27.7% in Westphalia-Lippe, and 46.8% in Schleswig-Holstein (see table 1 (Tab. 1)).

### 3.2. Practical clinical skills

#### 3.2.1. Clinical examination

**Figure 1:**
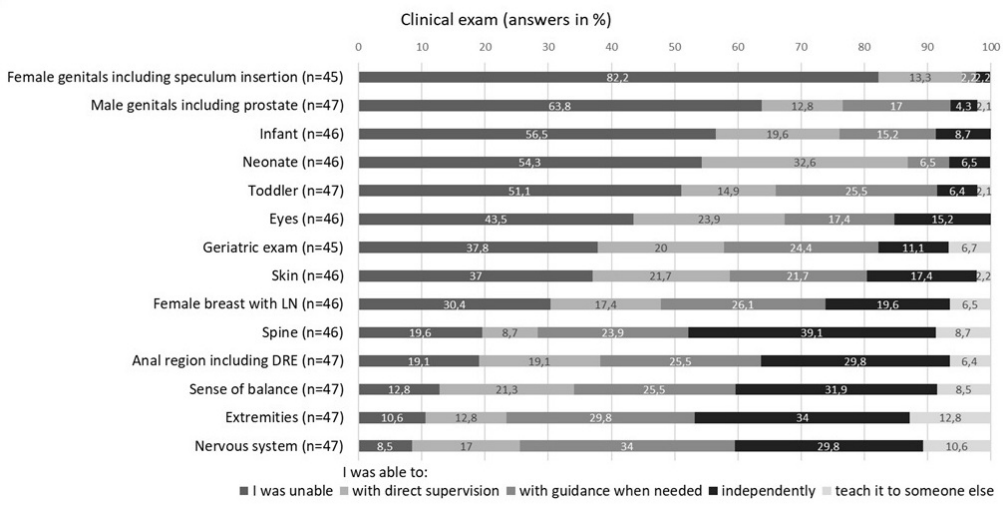
Clinical exam (license ≤5 years) Note: LN= lymph nodes; DRE= digital-rectal exam

Regarding skills required to clinically examine an adult patient, the majority of respondents (82.2%, n=37) reported that they had been “unable” to examine the female genitals including speculum insertion. More than half of the participants stated that they had been unable to perform a physical exam on a newborn (54.3%, n=25), an infant (56.5%, n=26), or to examine the male genitals including a prostate check (63.8%, n=30). Assessing sense of balance and spatial orientation was evaluated by 32% of the participants as something they could do on their own (n=15) and by 8.5% as something they could teach someone else (n=4). An overview is provided in figure 1 (Fig. 1).

#### 3.2.2. Skin allergy test

The majority of respondents reported being unable to perform all three of the skin tests surveyed: a skin prick test (76.7%, n=33), intracutaneous test (90.7%, n=39), and epicutaneous test (83.7%, n=36).

#### 3.2.3. Other practical clinical skills

**Figure 2:**
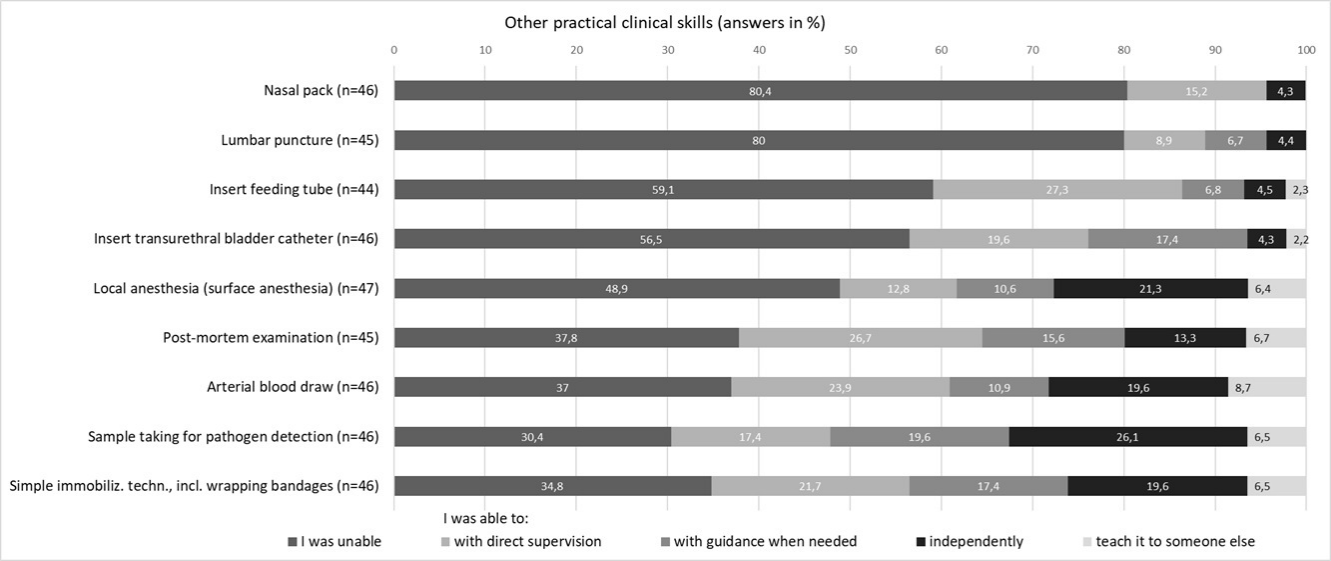
Other practical clinical skills (license ≤5 years)

Placing a nasal pack (80.4%, n=37) and performing a lumbar puncture (80.0%, n=36) were not skills that the majority felt they had mastered. More than half of the residents reported that they were not proficient in transurethral bladder catheterization (56.5%, n=26) or feeding tube insertion (59.1%, n=26). A total of 37.0% (n=17) stated that they had been unable to perform an arterial blood draw (see figure 2 (Fig. 2)).

#### 3.2.4. Administering medication with consideration of the benefits, downsides, and particularities of different injection sites

**Figure 3:**
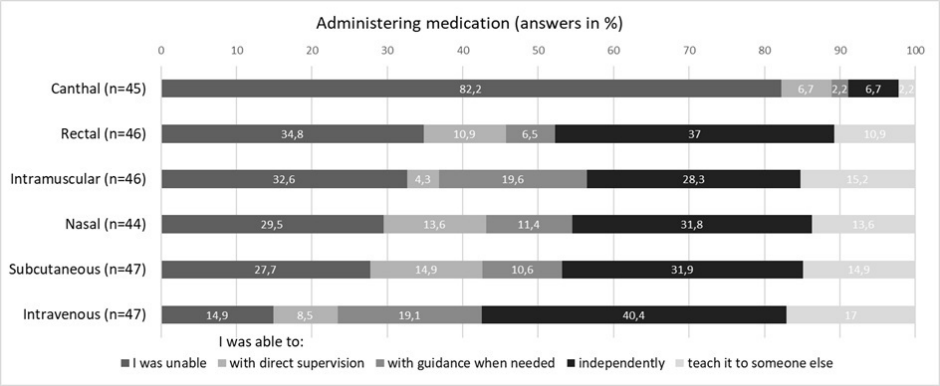
Figure 3 : Administering medication (license ≤5 years)

Regarding these practical clinical skills, the majority responded that they had been able to perform them independently or that they could have taught someone else how to do them: 57.4% (n=27) stated they had been able to administer medication intravenously, 46.8% (n=22) subcutaneously, and 47.9% (n=22) rectally. Only canthal administration was reported by the majority (82.2%, n=37) as something they had been unable to perform (see figure 3 (Fig. 3)).

#### 3.2.5. Practical communication skills

For the communication skills surveyed, the majority stated that they had mastered the explanation and demonstration of age-appropriate insertion of an IV line (46.8%, n=22) or that they could have taught somebody else how to do this (31.9%, n=15). In contrast, 63.8% (n=30) indicated they had been unable to explain or demonstrate the therapeutic use of inhalers and nebulizers in children. A total of 37.0% (n=17) reported that they had been unable to ascertain and document if a patient was at risk for harming themselves or others; 23.9 % of the participants (n=11) reported having been able to do this independently, and another 23.9 % indicated that they could have done this with supervision as needed. Compiling a psychopathological report was not something 26.1% (n=12) of the participants had been able to do, 26.1% (n=12) could do it under direct supervision, 37.0% (n=17) under indirect supervision, and 8.7% (n=4) independently.

### 3.3. Location where competence was acquired

On average, 88.3% of the participants identified undergraduate medical school as the place where they had acquired their skills (see table 2 (Tab. 2)).

### 3.4. Sub-analysis of the expanded sample

**Figure 4:**
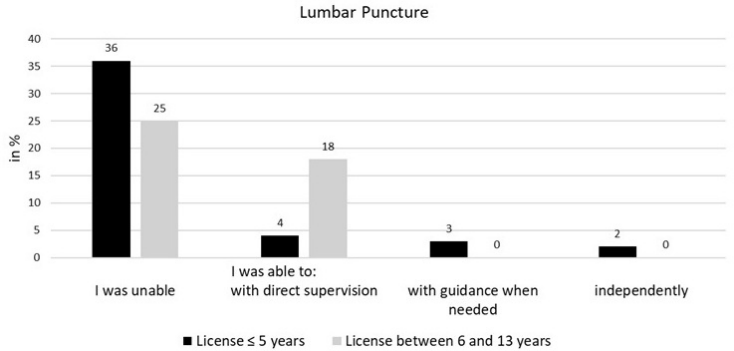
Performing a lumbar puncture (license ≤5 years vs. 6-13 years)

After Bonferroni correction, the chi-squared tests carried out in the sub-analysis showed a difference only between group 1 (license ≤5 years) and group 2 (license 6-13 years) in respect to performing a lumbar puncture. In the cross-tabulation four cells have an expected cell frequency lower than 5. There was a statistically significant correlation between both groups, χ^2^(1)=15.8555, p<0.001, φ=0.42 (see figure 4 (Fig. 4)).

Since the two groups differed significantly only in their proficiency in lumbar punction, the response frequencies for all participants are described in attachment 2 to give an expanded overview. Here, a similar response pattern is seen overall. For 31 of 36 skills, at least 20% of the participants stated that they had not yet mastered them at the start of their postgraduate training. This involved the same skills as for the residents whose license was ≤5 years old, with the exception of examination of the anal region (23.1% vs. 19.1% unable) and the spine (20.7% vs. 19.6% unable); see attachment 2 also.

## 4. Discussion

Based on a sample of medical residents (from the postgraduate competence centers in North Rhine, Westphalia-Lippe and Schleswig-Holstein) who self-assessed their competency levels at the beginning of their primary care residency training, this study provides information about their perceived proficiency at certain practical clinical skills from the NKLM 2.0. Drawn from a relatively small sample, the results suggest that a significant percentage of participants had not been able to perform most of the skills at the competency level envisioned by the current NKLM at the beginning of postgraduate training. By the end of undergraduate study (meaning during the final practical year of medical school), medical students should be able to perform most of the skills indicated in the NKLM for this survey under direct supervision (3a); only for some of the skills is the standard set at independent performance (3b). For 29 of the 36 practical clinical skills surveyed, at least every fifth resident stated that they had been unable to perform it; for 14 of these, more than half of the participants felt unable to perform the skill. Among these were basic skills such as examining the spine of an adult *(NKLM: 3b)* which one out of every five participants reportedly could not have performed. Still more rare was proficiency at examinations of male and female genitalia *(NKLM: 3a)* and at the physical examination of a child *(NKLM: 3a)*. In contrast, the majority of respondents claimed to have mastered technical skills such as an arterial blood draw and age-appropriate insertion of an IV line by the beginning of their residency training. On the one hand, this different level of proficiency could be because skills such as placing an IV line belong to the typical tasks in clinical electives and the final practical year; on the other hand, it could be that students have a higher intrinsic interest in being able to perform such tasks.

All in all, our results appear to corroborate the findings of previous studies in which physician trainees were unable to independently perform common procedures [7], [8]. Even in a voluntary sample of 214 final-year medical students performing objective structured clinical examination, deficits were discovered when carrying out practical clinical skills such as a physical exam [19], whereas other studies of physician trainees found self-confidence in conducting clinical exams, yet a lack of confidence in treating chronically ill patients or conducting early detection screening [20].

Our results may reveal a potential for improvement in the teaching of practical clinical skills.

In addition to spatial limitations, possible reasons for these identified gaps may be the insufficient focus on skills and practice in medical education and the associated lack of training opportunities [21]. Medical schools should ensure students master clinical skills during undergraduate medical education. Thereby, medical schools would create more opportunities so that basic skills, such as exams of the skin or an adult spine, can be performed independently by all graduates (3b as per NKLM) and so that other skills, such as pediatric exams, can be carried out under guidance by more than only half of the graduates. That said, these practical clinical skills represent only part of a competency-based education. Competency comprises not just skills but also of knowledge and behavior, applied in the clinical context for the wellbeing of the patient [22]. Under these circumstances, a stronger emphasis on practice and a focus on competency-based teaching and learning in undergraduate medical education may be needed. This should be achieved in future through, among other things, more clinical rotations, bed-side teaching, and simulation- based teaching [2], [23]. In addition, clinical traineeships mentored by university teaching staff may be another approach [24]. Relatedly, appropriate teaching and feedback methods in university teaching should be established [25], e.g., the implementation of *entrustable professional activities (EPA)* [26] and *workplace-based assessments* [27] (e.g., *Mini-Clinical Evaluation Exercise (Mini-CEX)* [28] or *Direct Observed Practical Skills (DOPS)* [29]). According to a survey of medical school teachers, these concepts and strategies should be expanded and further developed [30].

Moreover, the existing catalogue of learning objectives may be in part too extensive (e.g., performing skin allergy tests, lumbar punctures, and arterial blood draws, all at competency level 3a) and should be more focused on basic skills, such as clinical examination, e.g., of the musculoskeletal system. A clearer delineation of the learning objectives could possibly decrease the discrepancy felt by so many newly licensed physicians between what they learned at the university and what they can apply independently in their practice [31]. This aspect is already being considered in the current comprehensive revision of the NKLM, particularly regarding the desired competency level at the end of medical school vs. skills to be learned during residency, and with a reduction in the number of learning objectives.

### 4.1. Strengths and limitations

Our sample was mostly female, and the average participant age was 34 years. The sample can therefore be considered representative in terms of age and gender distribution for physician trainees undergoing primary care training [32], [33]. Another strength is that residents from the more rural Schleswig-Holstein and from the predominantly densely populated state of North Rhine-Westphalia were surveyed, which further contributes to the representativeness of the data.

However, all participants came from postgraduate competence centers, making it impossible to rule out a selection bias toward particularly committed residents with certainty.

The method of self-assessment was chosen due to its feasibility and cost-effectiveness. This could have led to limitations in the validity of our results. The subjectivity of the residents must be taken into consideration as an influencing factor, e.g., concerning the ability to critically reflect on one's own competency level.

The responding residents were mostly in the fourth or fifth year of postgraduate training with a wide age range; therefore, in terms of a recall bias, they may not have been able to remember exactly what their level of proficiency had been or where they had acquired their competency. Possibly, participants self-assessed their competency level as too low and marked a large portion of the skills as ones they had not been able to do. Juxtaposed to this is the observation that residents at a later stage of postgraduate training are better than beginning residents at evaluating and assesing their competency level and at which point in time skills were learned [8], [19].

To address the limitations identified above, future studies should compare the self-assessment of students at the end of medical school with an outside evaluation regarding competency levels in practical clinical skills and their application in the clinical context.

Although our study is limited by the fact that half of the participants were at an advanced stage of postgraduate training and thus also had a longer period of time since first entering medical practice, comparison between residents who had been licensed within the past five years with those licensed between six and 13 years ago showed no significant differences.

Our sample size does not allow any generalized statements. However, the results appear to concur with previous studies [7], [8], [19], so that it can be assumed that the findings are not diminished by the low number of participants.

## 5. Conclusion

The present study points to a potential discrepancy between the competency level perceived by medical residents and the desired level of competency during undergraduate medical education as specified in the NKLM 2.0. Residents may need to make up ground in respect to the learning of particular practical clinical skills. Since such skills form only one part of a competency-based education, the required educational changes resulting from a stronger emphasis on competency in undergraduate medical education could be extremely broad and comprehensive. Hence, medical schools need to create the conditions under which a range of needs, from appropriate teaching spaces to simulated patients and skills labs, can be met and instructors are enabled to fulfill teaching duties.

## Authors’ ORCIDs

- Dorothea Dehnen: [0000-0002-1562-7178]
- Kristina Flägel: [0000-0002-1416-6293]
- Dorothea Wild: [0000-0001-9410-1766]
- Jost Steinhäuser: [0000-0002-9386-6078]

## Acknowledgements

The authors wish to thank Prof. Dr. Bert Huenges, who facilitated the survey at the Westphalia-Lippe Competence Center (KWWL) and critically commented on a draft of the paper, and Katja Maercklin for organizing the survey at KWWL.

## Competing interests

The authors declare that they have no competing interests. 

## Supplementary Material

Questionnaire

Supplementary table

## Figures and Tables

**Table 1:**
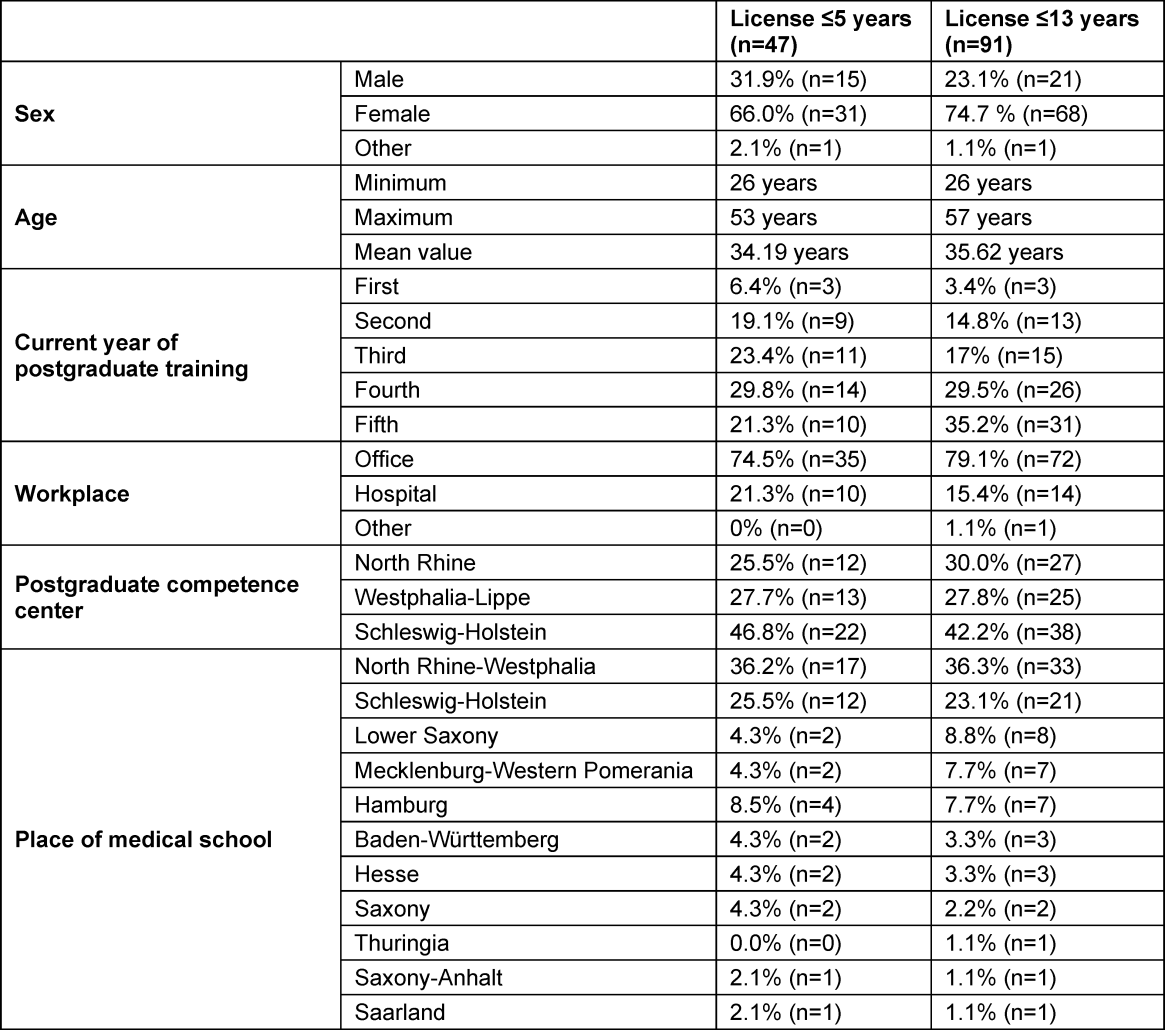
Sociodemographic profile of the participants

**Table 2:**
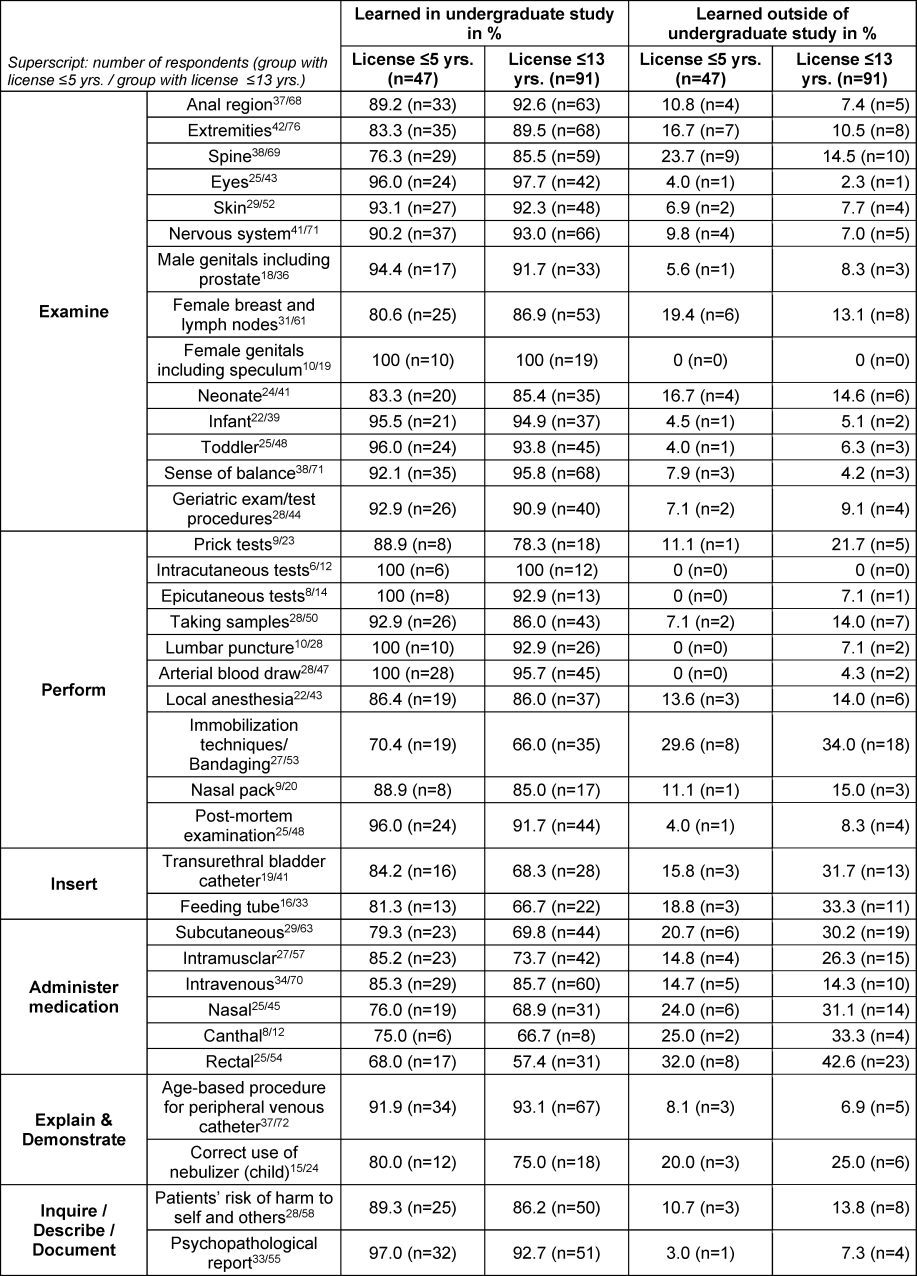
Location where competency was acquired (This question was not asked of a particular respondent if their response to the question about proficiency was “I was unable to do this”)
