# Supplementary material for: To what extent have national learning objectives in undergraduate medical education been achieved? A cross-sectional study of primary care residents
Source: GMS J Med Educ. 2025 Jun 16;42(3):Doc38. doi: 10.3205/zma001762 (PMC12286870; doi:10.3205/zma001762)
Supplement: Questionnaire [file JME-42-38-s-001.pdf]

Attachment 1: Questionnaire

Attachment 1 to Dehnen D, Flägel K, Wild D. Steinhäuser J. *To what extent have national learning objectives in undergraduate medical education been achieved? A cross-sectional study of primary care residents*. GMS J Med Educ. 2025;42(3):Doc38. DOI: 10.3205/zma001762

To what extent have national learning objectives in undergraduate medical education been achieved? A cross-sectional study of primary care residents

Dear colleagues,

We would like to invite you to participate in a study investigating the above question.

In April 2021, the new National Competency-based Catalogue of Learning Objectives for Undergraduate Medical Education 2.0 (NKLM 2.0) was published. The catalogue of learning objectives defines the competencies in which every physician should be proficient upon graduation from medical school. In addition to knowledge and clinical skills, there are also broader learning objectives such as professional conduct and attitude, academic scholarship, and "soft skills."[1,2] This study will investigate the extent to which selected medical skills in the NKLM 2.0 have been mastered and are applied as perceived by postgraduate physician trainees. By participating in this study, you contribute to improving medical education!

This study entails an **anonymous** survey from which it is impossible to draw any conclusions about you or any other person. No registration is necessary to participate in the study. Any participation in the study is **voluntary**!

Should you have any questions about this study, please contact Dr. Dorothea Dehnen at dorothea.dehnen@uk-essen.de, 0201/723-8036

Thank you for participating. You can begin the survey here:

1. Medizinischer Fakultätentag. Kompetenzbasierte Lernzielkataloge (NKLM, NKLZ) – aus den Fakultäten und für die Fakultäten. Online publication: https://medizinische-fakultaeten.de/themen/aktuelle-themen/nklm-nklz/
2. Medizinischer Fakultätentag: Nationaler kompetenzbasierter Lernzielkatalog Medizin – Version 2.0. NKLM Loop Projekt Charité. Online publication: https://nklm.de/zend/objective/list/orderBy/@objectivePosition/studiengang/Info

Please think back to the beginning of your first year of postgraduate training and think about whether you had learned the following skills during medical school or elsewhere (e.g., as part of previous professional training) and which level of competency you had attained at that time (you will need to make two marks per line). This refers to the competency level in treating an average patient in a general practitioner's office:

	**I was unable.**	**I was able to:**				**Where was skill acquired?**	
	If true, then proceed to next line.	**with direct supervision**	**with guidance when needed**	**independently**	**teach it to someone else**	**During regular study (including clinical electives)**	**Outside of medical school (e.g., prior training, practicum prior to medical school, etc.)**
**Clinical examination of:**							
Anal region, including digital-rectal exam							
Extremities							
Spine							
Eyes							
Skin and skin appendages							
Nervous system							
Male genitals including prostate							
Female breast and lymph nodes							
Neonate (e.g., gestational age, heart function, respiratory function, malformations, APGAR score)							
Infant (general and disease-specific)							

Attachment 1 to Dehnen D, Flägel K, Wild D. Steinhäuser J. *To what extent have national learning objectives in undergraduate medical education been achieved? A cross-sectional study of primary care residents*. GMS J Med Educ. 2025;42(3):Doc38. DOI: 10.3205/zma001762

	I was **unable**.	I was able to:				Where was skill acquired?	
	If true, then proceed to next line.	**with direct supervision**	**with guidance when needed**	**independently**	**teach it to someone else**	**During regular study (including clinical electives)**	**Outside of medical school (e.g., prior training, practicum prior to medical study, etc.)**
Toddler (general and disease-specific)							
**Performance of:**							
Exam to assess sense of balance and spatial orientation							
Examination of female genitals including use of speculum							
Geriatric exam & test procedures							
Prick test							
Intracutaneous test							
Epicutaneous test							
Correct sample taking for pathogen detection							
Lumbar puncture							
**Arterial** blood draw							
Local anesthesia (surface)							
Basic immobilization techniques/Bandaging							
Nasal pack							
Post-mortem examination							

	I was unable.	I was able to:				Where was skill acquired?	
	If true, then proceed to next line.	with direct supervision	with guidance when needed	idependently	teach it to someone else	During regular study (including clinical electives)	Outside of medical school (e.g., prior training, practicum prior to medical study, etc.)
**Insertion of:**							
Transurethral bladder catheter							
Feeding tube							
**Administration of medication with consideration of the benefits, drawbacks and particularities of different injection sites:**							
Subcutaneous							
Intramuscular							
Intravenous							
Nasal							
Canthal							
Rectal							
**Explanation and demonstration of:**							
Age-specific procedure to place an IV line							
Correct use of inhalers and nebulizers in children							
**Inquire about: / Describe and document:**							
Patients' risk of harm to self and others							
Psychopathological report							

UNIVERSITÄT ZU LÜBECK
INSTITUT FÜR ALLGEMEINMEDIZIN

Universitätsmedizin Essen
Universitätsklinikum

ifam
Institut für Allgemeinmedizin

In closing, we would like to request that you share some information about yourself.

Personal information

Age: ______________________________

Gender: ☐ Female ☐ Male ☐ Other

Year of licensure: ______________________________

Location of the medical school you attended *(Please indicate the medical school where you completed the majority of your undergraduate study)*: ______________________________

Current year of postgraduate training: 1 ☐ 2 ☐ 3 ☐ 4 ☐ 5 ☐

Which postgraduate competence center: North Rhine (KWNO) ☐ Westphalia-Lippe (KWWL) ☐ Schleswig-Holstein (KWA SH) ☐

Have you completed training in another medical specialty?: ☐ Yes ☐ No

If so, which specialty? ______________________________

Do you already have an additional qualification?: ☐ Yes ☐ No

If so, which qualification? ______________________________

Currently working at: ☐ Office ☐ Hospital ☐ On parental leave ☐ Other: ______________________________

Attachment 1 to Dehnen D, Flägel K, Wild D. Steinhäuser J. *To what extent have national learning objectives in undergraduate medical education been achieved? A cross-sectional study of primary care residents*. GMS J Med Educ. 2025;42(3):Doc38. DOI: 10.3205/zma001762
