# Supplementary material for: To what extent have national learning objectives in undergraduate medical education been achieved? A cross-sectional study of primary care residents
Source: GMS J Med Educ. 2025 Jun 16;42(3):Doc38. doi: 10.3205/zma001762 (PMC12286870; doi:10.3205/zma001762)
Supplement: Supplementary table [file JME-42-38-s-002.pdf]

Attachment 2: Supplementary table

Table S1: Competency level compared by group

Superscript: number of respondents (group with license ≤ 5 yrs. / group with license ≤ 13 yrs.)	**I was able to:**								**I was unable to do it.** (highlighted in gray if >50%)	
	**teach it to someone else [%]**		**independently [%]**		**with guidance when needed [%]**		**with direct supervision [%]**			
	**Lic. ≤ 5 yrs.**	**Lic. ≤ 13 yrs.**	**Lic. ≤ 5 yrs.**	**Lic. ≤ 13 yrs.**	**Lic. ≤ 5 yrs.**	**Lic. ≤ 13 yrs.**	**Lic. ≤ 5 yrs.**	**Lic. ≤ 13 yrs.**	**Lic. ≤ 5 yrs.**	**Lic. ≤ 13 yrs.**
**Examine**										
**Anal region**[47/91]	6.4 (n=3)	6.6 (n=6)	29.8 (n=14)	29.7 (n=27)	25.5 (n=12)	26.4 (n=24)	19.1 (n=9)	14.3 (n=13)	19.1 (n=9)	23.1 (n=21)
**Extremities**[47/89]	12.8 (n=6)	7.9 (n=7)	34.0 (n=16)	33.7 (n=30)	29.8 (n=14)	37.1 (n=33)	12.8 (n=6)	10.1 (n=9)	10.6 (n=5)	11.2 (n=10)
**Spine**[46/87]	8.7 (n=4)	5.7 (n=5)	39.1 (n=18)	33.3 (n=29)	23.9 (n=11)	29.9 (n=26)	8.7 (n=4)	10.3 (n=9)	19.6 (n=9)	20.7 (n=18)
**Eyes**[46/88]	/	1.1 (n=1)	15.2 (n=7)	13.6 (n=12)	17.4 (n=8)	13.6 (n=12)	23.9 (n=11)	21.6 (n=19)	43.5 (n=20)	50.0 (n=44)
**Skin**[46/89]	2.2 (n=1)	1.1 (n=1)	17.4 (n=8)	14.6 (n=13)	21.7 (n=10)	25.8 (n=23)	21.7 (n=10)	20.2 (n=18)	37.0 (n=17)	38.2 (n=34)
**Nervous system**[47/89]	10.6 (n=5)	6.7 (n=6)	29.8 (n=14)	27.0 (n=24)	34.0 (n=16)	39.3 (n=35)	17.0 (n=8)	14.6 (n=13)	8.5 (n=4)	12.4 (n=11)
**Male genitals including prostate**[47/90]	2.1 (n=1)	2.2 (n=2)	4.3 (n=2)	4.4 (n=4)	17.0 (n=8)	18.9 (n=17)	12.8 (n=6)	15.6 (n=14)	63.8 (n=30)	58.9 (n=53)
**Female breast and lymph nodes**[46/90]	6.5 (n=3)	3.3 (n=3)	19.6 (n=9)	20.0 (n=18)	26.1 (n=12)	23.3 (n=21)	17.4 (n=8)	23.3 (n=21)	30.4 (n=14)	30.0 (n=27)
**Female genitals including speculum**[45/87]	/	/	2.2 (n=1)	1.1 (n=1)	2.2 (n=1)	1.1 (n=1)	13.3 (n=6)	17.2 (n=15)	82.2 (n=37)	80.5 (n=70)
**Neonate**[46/88]	/	/	6.5 (n=3)	6.8 (n=6)	6.5 (n=3)	9.1 (n=8)	32.6 (n=15)	28.4 (n=25)	54.3 (n=25)	55.7 (n=49)
**Infant**[46/88]	/	/	8.7 (n=4)	4.5 (n=4)	15.2 (n=7)	17.0 (n=15)	19.6 (n=9)	20.5 (n=18)	56.5 (n=26)	58.0 (n=51)
**Toddler**[47/90]	2.1 (n=1)	1.1 (n=1)	6.4 (n=3)	4.4 (n=4)	25.5 (n=12)	23.3 (n=21)	14.9 (n=7)	25.6 (n=23)	51.1 (n=24)	45.6 (n=41)
**Sense of balance**[47/90]	8.5 (n=4)	5.6 (n=5)	31.9 (n=15)	40.0 (n=36)	25.5 (n=12)	22.2 (n=20)	21.3 (n=10)	15.6 (n=14)	12.8 (n=6)	16.7 (n=15)
**Geriatric exam & test procedures**[45/88]	6.7 (n=3)	3.4 (n=3)	11.1 (n=5)	14.8 (n=13)	24.4 (n=11)	20.5 (n=18)	20.0 (n=9)	12.5 (n=11)	37.8 (n=17)	48.9 (n=43)
**Perform**										
**Prick tests**[43/86]	2.3 (n=1)	1.2 (n=1)	7.0 (n=3)	7.0 (n=6)	7.0 (n=3)	9.3 (n=8)	7.0 (n=3)	11.6 (n=10)	76.7 (n=33)	70.9 (n=61)
**Intracutaneous tests**[43/84]	/	/	2.3 (n=1)	3.6 (n=3)	/	3.6 (n=3)	7.0 (n=3)	6.0 (n=5)	90.7 (n=39)	86.9 (n=73)

Attachment 2 to Dehnen D, Flägel K, Wild D. Steinhäuser J. *To what extent have national learning objectives in undergraduate medical education been achieved? A cross-sectional study of primary care residents*. GMS J Med Educ. 2025;42(3):Doc38. DOI: 10.3205/zma001762

**Epicutaneous tests[43/85]**	/	/	4.7 (n=2)	4.7 (n=4)	/	4.7 (n=4)	11.6 (n=5)	7.1 (n=6)	83.7 (n=36)	83.5 (n=71)
**Taking samples for pathogen detection[46/88]**	6.5 (n=3)	6.8 (n=6)	26.1 (n=12)	25.0 (n=22)	19.6 (n=9)	14.8 (n=13)	17.4 (n=8)	17.0 (n=15)	30.4 (n=14)	36.4 (n=32)
**Lumbar puncture[45/88]**	/	/	4.4 (n=2)	2.3 (n=2)	6.7 (n=3)	3.4 (n=3)	*8.9 (n=4)*	*25.0 (n=22)*	*80.0 (n=36)*	*69.3 (n=61)*
**Arterial blood draw[46/89]**	8.7 (n=4)	7.9 (n=7)	19.6 (n=9)	18.0 (n=16)	10.9 (n=5)	13.5 (n=12)	23.9 (n=11)	20.2 (n=18)	37.0 (n=17)	40.4 (n=36)
**Local anesthesia[47/89]**	6.4 (n=3)	5.6 (n=5)	21.3 (n=10)	16.9 (n=15)	10.6 (n=5)	14.6 (n=13)	12.8 (n=6)	16.9 (n=15)	48.9 (n=23)	46.1 (n=41)
**Immobilization techniques/Bandaging[46/8]**	6.5 (n=3)	8.0 (n=7)	19.6 (n=9)	21.6 (n=19)	17.4 (n=8)	13.6 (n=12)	21.7 (n=10)	21.6 (n=19)	34.8 (n=16)	35.2 (n=31)
**Nasal pack[46/88]**	/	/	4.3 (n=2)	6.8 (n=6)	/	4.5 (n=4)	15.2 (n=7)	12.5 (n=11)	80.4 (n=37)	76.1 (n=67)
**Post-mortem examination[45/89]**	6.7 (n=3)	4.5 (n=4)	13.3 (n=6)	14.6 (n=13)	15.6 (n=7)	20.2 (n=18)	26.7 (n=12)	21.3 (n=19)	37.8 (n=17)	39.3 (n=35)
**Insert**										
**Transurethral bladder catheter[46/89]**	2.2 (n=1)	6.7 (n=6)	4.3 (n=2)	12.4 (n=11)	17.4 (n=8)	11.2 (n=10)	19.6 (n=9)	19.1 (n=17)	56.5 (n=26)	50.6 (n=45)
**Feeding tube[44/84]**	2.3 (n=1)	4.8 (n=4)	4.5 (n=2)	7.1 (n=6)	6.8 (n=3)	7.1 (n=6)	27.3 (n=12)	26.2 (n=22)	59.1 (n=26)	54.8 (n=46)
**Administer medication**										
**Subcutaneous[47/89]**	14.9 (n=7)	18.0 (n=16)	31.9 (n=15)	41.6 (n=37)	10.6 (n=5)	5.6 (n=5)	14.9 (n=7)	13.5 (n=12)	27.7 (n=13)	21.3 (n=19)
**Intramuscular[46/88]**	15.2 (n=7)	13.6 (n=12)	28.3 (n=13)	28.4 (n=25)	19.6 (n=9)	19.3 (n=17)	4.3 (n=2)	9.1 (n=8)	32.6 (n=15)	29.5 (n=26)
**Intraveneous[47/91]**	17.0 (n=8)	19.8 (n=18)	40.4 (n=19)	46.2 (n=42)	19.1 (n=9)	14.3 (n=13)	8.5 (n=4)	6.6 (n=6)	14.9 (n=7)	13.2 (n=12)
**Nasal[44/85]**	13.6 (n=6)	9.4 (n=8)	31.8 (n=14)	35.3 (n=30)	11.4 (n=5)	8.2 (n=7)	13.6 (n=6)	9.4 (n=8)	29.5 (n=13)	37.6 (n=32)
**Canthal[45/84]**	2.2 (n=1)	1.2 (n=1)	6.7 (n=3)	4.8 (n=4)	2.2 (n=1)	2.4 (n=2)	6.7 (n=3)	4.8 (n=4)	82.2 (n=37)	86.9 (n=73)
**Rectal[46/90]**	10.9 (n=5)	10.0 (n=9)	37.0 (n=17)	43.3 (n=39)	6.5 (n=3)	8.9 (n=8)	10.9 (n=5)	8.9 (n=8)	34.8 (n=16)	28.9 (n=26)
**Explain and Demonstrate**										
**Age-specific peripheral venous catheter[47/91]**	31.9 (n=15)	26.4 (n=24)	46.8 (n=22)	50.5 (n=46)	2.1 (n=1)	4.4 (n=4)	8.5 (n=4)	6.6 (n=6)	10.6 (n=5)	12.1 (n=11)
**Correct use of nebulizer in child[47/88]**	4.3 (n=2)	4.5 (n=4)	10.6 (n=5)	10.2 (n=9)	10.6 (n=5)	8.0 (n=7)	10.6 (n=5)	8.0 (n=7)	63.8 (n=30)	69.3 (n=61)
**Inquire / Describe / Document**										
**Patients' risk of harm to self and others[6/88]**	8.7 (n=4)	5.7 (n=5)	23.9 (n=11)	25.0 (n=22)	23.9 (n=11)	29.5 (n=26)	6.5 (n=3)	10.2 (n=9)	37.0 (n=17)	29.5 (n=26)
**Psychopathological report[46/89]**	2.2 (n=1)	1.1 (n=1)	8.7 (n=4)	11.2 (n=10)	37.0 (n=17)	32.6 (n=29)	26.1 (n=12)	20.2 (n=18)	26.1 (n=12)	34.8 (n=31)

Attachment 2 to Dehnen D, Flägel K, Wild D. Steinhäuser J. *To what extent have national learning objectives in undergraduate medical education been achieved? A cross-sectional study of primary care residents*. GMS J Med Educ. 2025;42(3):Doc38. DOI: 10.3205/zma001762
